# New observations in the fragile X-associated tremor/ataxia syndrome (FXTAS) phenotype

**DOI:** 10.3389/fgene.2014.00365

**Published:** 2014-10-17

**Authors:** Avram Fraint, Padmaja Vittal, Aimee Szewka, Bryan Bernard, Elizabeth Berry-Kravis, Deborah A. Hall

**Affiliations:** ^1^Department of Neurological Sciences, Rush UniversityChicago, IL, USA; ^2^Department of Pediatrics, Neurological Sciences and Biochemistry, Rush UniversityChicago, IL, USA

**Keywords:** FXTAS, *FMR1*, FXS, CGG, premutation

## Abstract

**Purpose:** Fragile X-associated tremor/ataxia syndrome (FXTAS) was originally defined as tremor, ataxia, cognitive decline, and parkinsonism in individuals who carry between 55 and 200 CGG repeats in the promoter region of the *fragile X mental retardation 1* (*FMR1*) gene. This paper describes a series of patients who meet the definition of FXTAS who presented for care between 2009 and 2014.

**Methods/Results:** Retrospective chart review of patients seen in the FXTAS clinic at Rush University in Chicago.

**Conclusions:** Patients with FXTAS may present with a progressive supranuclear palsy-like phenotype and other eye movement abnormalities are common in these patients as well. Rapid worsening of gait abnormalities in FXTAS may be due to a secondary spinal issue and should be aggressively treated to regain function. Finally, the FXTAS Rating Scale score does not reliably inform the certainty of diagnosis or CGG repeat size in these patients.

## Introduction

Fragile X-associated tremor/ataxia syndrome (FXTAS) is caused by expansion in the trinucleotide CGG repeat in the promoter region of the *fragile X mental retardation 1* (*FMR1)* gene. Classical clinical manifestations include kinetic tremor, cerebellar ataxia, cognitive decline, psychiatric problems, and parkinsonism (Jacquemont et al., 2003, 2004; Berry-Kravis et al., 2007a). Other features include peripheral neuropathy (Berry-Kravis et al., 2007b), impotence and autonomic dysfunction (including bowel and/or bladder dysfunction and erectile dysfunction) (Louis et al., 2006; Leehey, 2009). Cognitive decline manifests as a frontal subcortical dementia, with memory loss and executive function deficits and often lack of insight into these deficits (Grigsby et al., 2007). Psychiatric effects of FXTAS include anxiety, mood lability, apathy, and social phobias (Bourgeois et al., 2006). It is most frequently seen in individuals over the age of fifty who carry between 55 and 200 CGG repeats, also known as the “premutation.” Fragile X syndrome (FXS), the most common inherited form of intellectual disability, results from the presence of more than 200 CGG repeats and is characterized by intellectual disability, autism, attention deficit disorder, and often seizures.

FXTAS has distinct features on magnetic resonance imaging (MRI), including severe generalized atrophy, cerebellar atrophy, and sub-cortical and/or ponto-cerebellar white matter lesions (Greco et al., 2006). About 60% of males with FXTAS have what is known as the “MCP sign,” or T2 hyperintensity in the middle cerebellar peduncle (Adams et al., 2007). Hyperintensities in the splenium of the corpus callosum on MRI may also be seen (Apartis et al., 2012).

The estimated prevalence of the *FMR1* premutation is between 1/151 and 1/209 in women and 1/430—1/468 in men (Seltzer et al., 2012; Tassone et al., 2012b). Since its initial description, more women with FXTAS are being identified. Some medical comorbidities may be more common in premutation carrier women, including thyroid disease, hypertension, seizures, and fibromyalgia (Coffey et al., 2008; Leehey et al., 2011). The purpose of this project is to demonstrate the heterogeneity in patients presenting for clinical care of FXTAS.

## Background

Thirty patients with FXTAS were seen between 2009 and 2014 for clinical care in the FXTAS Clinic. Nineteen cases with complete clinical information were summarized for this study. Their clinical characteristics are described in Table 1. FXTAS Motor Rating Scale scores are listed in the table and encompass the major movement disorder signs seen in FXTAS: tremor, ataxia, and parkinsonism. This rating scale was developed in 2008. It is a combination of the Clinical Rating Scale for Tremor (CRST), the International Cooperative Ataxia Rating Scale (ICARS) and the Unified Parkinson's Disease Rating Scale (UPDRS). It also includes a tandem test for cerebellar gait ataxia (Leehey et al., 2008). Four cases illustrative of the series have been included.

**Table 1:** **Clinical features of FXTAS cases**.

**Case**	**Age**	**Sex**	*FMR1* premutation size^*^	**Diagnosis of FXTAS**	**Tremor**	**Ataxia**	**Nystagmus or other eye movement abnormality**	**Neuropathy symptoms or signs**	**Cognition**	**Neuropsychiatric**	**FXTAS rating scale score**	**MRI**	**Family history of fragile X disorders**	**Other features**
1	56	M	150	Definite	Mild	Severe	Saccadic pursuits	Diminished vibration and proprioception in feet	MMSE 11/30, moderate severe dementia	Aggressive behavior	87	White matter hyperintensities, moderate diffuse volume loss, left frontal mass	Grandson and grandnephew with FXS	Metatstatic brain cancer
2^**^	75	M	124	Definite	Severe	Mild	No vertical OKN, lateral endgaze nystagmus, decreased lateral OKN	Decreased vibration in feet	18/30 MMSE,	Apathy, withdrawn socially	73	Severe non-specific white matter changes, midbrain volume loss, MCP sign	Two nieces with FXS	Monoclonal Gammopathy of Unknown Significance
3	62	F	23, 120	Possible	No	Mild	Horizontal endgaze nystagmus	No	29/30 MMSE, deficit in encoding new information	Depression	7	Mild cerebral volume loss, scattered white matter changes	Daughter FXS	Severe fibromyalgia
4	69	F	26, 91	Definite	Mild	Moderate	Saccadic pursuits	No	MMSE 30/30	Irritability, auditory hallucinations, anxiety, depression	30	White matter changes periventricular and in pons	Grandson with FXS	Alcoholism, transient global amnesia
5	72	M	105	Probable	Mild	Mild		No	23/30 MOCA, deficits in executive function and recent memory	Poor insight	75	Global volume loss, MCP sign, basal ganglia calcifications	None	
6	46	F	20, 102	Possible	Mild	Mild	Lateral endgze nystagmus, saccadic pursuits	Decreased vibration in feet	MMSE 30/30	Mild depression	22	Mild cerebellar vermal volume loss	Daughter, three aunts, one uncle with FXS	Non-epileptic spells
7^**^	71	M	99	Definite	Moderate	Moderate	Dysmetric saccades	Vibratory loss in feet and temperature loss in limbs	24/30 MMSE, deficits in attention, executive function, recent memory	Agitation	51	Cerebral volume loss, hyperintensity ain cerebral peduncles, MCP sign	Sister, grandson, grandaughter with FXS, Mother with FXPOI	Myelodysplastic disorder, spinal cord mass
8	70	M	99	Definite	Moderate	Moderate	Saccadic pursuits	Decreased vibration in feet	MMSE 24/30, mild, generalized dementia	Depression, anger, apathy	64	MCP sign	Brother with intellectual disability, autism	Impotence, urinary issues
9^**^	79	F	29, 90	Definite	Mild	Mild	Saccadic pursuit, transient end gaze nystagmus	Decreased temperature, no reflexes	27/30 MMSE, mild deficit in recent memory, executive function, and language	Psychosis: delusions and visual hallucinations, agitation	47	Cerebral and cerebellar volume loss	Daughter, two nephews, and one niece with FXS	Vitiligo, diabetes, IgA deficiency, end stage renal disease
10	68	M	95	Possible	Mild	Mild	Normal	Length dependent sensory neuropathy	Cognition reported to be normal	No	38	Cerebral and cerebellar volume loss, lesion in cerebral peduncle (CT)	Sister with premature menopause	
11	70	M	91	Definite	Moderate	Mild	Dysmetric saccades	Decreased in stocking distribution	25/30 MMSE, deficits in executive function and recent memory	Disinhibition	72	MCP sign, scattered white matter ds	Niece and nephew with FXS	Achalasia
12^**^	75	F	88	Definite	Moderate	Moderate	No vertical OKN, slowed vertical saccades, +square wave jerks	Dec all modalities in feet	MMSE 25/30, deficits in executive function, recent memory, verbal fluency	Depression, apathy	69	Extensive white matter lesions in the pons and periventricular area	Sister with FXPOI, father and cousin with FXTAS	Apraxia, fibromyalgia, severe obstructive sleep apnea
13	72	M	85	Possible	Mild	Mild	Saccadic smooth pursuit vertically, hypometric vertical saccades, decreased convergence	Distal sensory loss feet, normal EMG/NCV	30/30 MMSE	Anxiety, irritability, subjective memory loss	41	Greater than age appropriate atrophy	Daughter and nephew with FXS, Father with possible FXTAS	Tinnitus, vertigo, dizziness; OSA
14	76	M	80	Probable	Moderate	Severe	Apraxia of eye movements, saccadic pursuits	Diminished reflexes at patella and ankle	27/30 MMSE, deficit in short-term memory	No	81	Not available	Grandson with FXS	Prostate and basal cell cancer
15	64	F	79	Probable	Mild	Mild	Normal	No	29/30 MMSE, deficit in executive function	Anxiety	27	Moderate volume loss, white matter hyperintensities	Two sons with FXS	Autonomic symptoms, bradycardia/tachycardia
16	80	M	75	Definite	Mild	Moderate	Saccadic pursuits	Decreased vibration in feet, absent reflexes at ankle	24/28 MMSE, deficits in attention and recent memory	Irritability	31	MCP sign	Grandnephew has FXS	
17	75	F	74	Definite	Mild	Mild	Overshoot, dysmetria of saccades, slowed vertical saccades with OKN, saccadic pursuits	Decreased reflexes in legs	29/30 MMSE	Irritability, impulsiveness	20	Scattered white matter hyperintensities	Two sons with FXS	Stage IV squamous cell lung cancer
18^**^	82	M	68	Probable	Moderate	Mild	Impaired vertical gaze and vertical OKN, slowed saccades	No	27/30 MMSE, frontal/subcortical dysfunction	Visual and auditory hallucinations	55	Ventriculomegaly (CT)	Grandson with FXS	REM Sleep Behavior Disorder
19	82	M	60	Probable	Mild	Mild	No	No	30/30 MMSE, deficit in short-term memory	None	17	Hyperintensities in left cerebellum, subcortex, global volume loss	Grandson with FXS	Hearing loss

* Premutation size only reported, mean of 94 with a range of 60–150;

** cases described in more depth in manuscript; OKN, optokinetic nystagmus, MMSE, Mini Mental Status Examination; FXS, fragile X syndrome; REM, rapid eye movement; FXPOI, fragile X associated primary ovarian insufficiency, MOCA, Montreal Cognitive Assessment

### Case 7

A 70 year-old man with history of myeloproliferative disorder presented with 3 years of balance problems and falls. He felt he was veering to one side when he was walking. He denied tremors and memory problems. On examination, he scored a 24/30 on the Folstein Mini Mental Status Exam (MMSE) (Folstein et al., 1975) with deficits in attention, recent memory, and executive function. He had dysmetric saccades. Sensory exam was notable for inconsistent vibratory loss in the feet and temperature loss in all extremities. He had mildly increased tone in both arms and mild kinetic tremor when drawing spirals. He had mild bradykinesia bilaterally. He had no evidence of dysmetria. His gait was wide based and ataxic. He was profoundly unstable when standing or walking, with almost immediate falling. He refused to use a walking aid. Brain MRI showed cerebral volume loss, hyperintensity in the cerebral peduncles, and the MCP sign. He was admitted due to a lack of safety with his balance and had a three-week rehabilitation stay. There he developed a sudden onset bilateral leg weakness. Workup revealed a large soft tissue mass in his spinal cord extending from T1 to T9 causing significant cord compression. It was resected and pathology was consistent with extramedullary hematopoeisis. He then underwent successful radiation therapy. After continued rehabilitation, he was able to regain prior function and is currently walking with a walker. His family history was remarkable for a sister and multiple grandchildren with FXS. His mother had fragile X-associated primary ovarian insufficiency (FXPOI). His *FMR1* CGG repeat size was 99. He met criteria for definite FXTAS, with tremor, ataxia, and the MCP sign on MRI.

### Case 9

A 75 year-old woman presented with a one-year history of balance problems, which she described as wobbling when she walked. She had fallen once. She denied tremors, but had decreased hearing in both ears, urinary frequency, and fatigue. Her past history was remarkable for diabetes, end stage renal disease, and vitiligo. On examination, she had normal cognition, saccadic pursuits, and transient endgaze nystagmus. She had increased tone in the right arm without cogwheel rigidity, anterocollis, and mild kinetic tremor with handwriting. Reflexes were absent in the extremities and sensation was decreased to temperature. Dysdiadochokinesia was present in the left hand and bradykinesia in the left leg. She was unable to stand or walk in tandem and got off balance when she turned quickly. There was no retropulsion on the pull test. Brain MRI showed cerebral and cerebellar volume loss with white matter hyperintensities in the periventricular region.

The following year, she had a fall and had surgery for a “pinched nerve” in the neck. She began to have visual hallucinations in the hospital and was started on haloperidol. Her examination had dramatically worsened: she was wheelchair bound and was no longer able to walk unless she had assistance. Her MMSE was 18/30. She then developed delusions and depression. Quetiapine and venlafaxine were added and haloperidol discontinued. She received aggressive inpatient and outpatient rehabilitation over the next 3 months and regained the ability to walk using a walker. Her *FMR1* CGG repeat sizes were 90 and 29. She had a daughter, two nephews, and one niece with FXS. She met criteria for definite FXTAS, with tremor, ataxia, and white matter disease on MRI.

### Case 12

A 72 year-old woman presented with progressive difficulty walking since 1998. She initially attributed this difficulty to pain in her feet, and was subsequently diagnosed with plantar fasciitis. By 2000–2001, she developed postural dizziness and soon developed a slow, festinating gait with decreased arm swing as well as fatigue, cognitive slowing, and trouble sleeping. By 2009 she was wheelchair bound. MRI of her brain revealed white matter lesions in the pons and periventricular regions. Testing for autoimmune disorders and spinocereballar ataxias (SCA) was negative. She failed treatment with amantadine and was started on carbidopa/levodopa 25–100 mg twice daily and her walking improved.

On examination at age 75, her MMSE was 25/30, with deficits in executive function, recent memory and verbal fluency. She had apraxia in her left hand and foot, a positive glabellar reflex, and trouble mimicking on the left side. She had absent vertical optokinetic nystagmus, slowed vertical saccades, and square wave jerks. She was bradykinetic and had resting tremor in both arms. Her gait was remarkable for short stride length, frequent freezing, difficulty with tandem gait and impaired postural reflexes. Her carbidopa-levodopa was weaned due to persistent nausea. Donepezil 10 mg daily was started given her cognitive decline. She continued frequent physical therapy at home, but her symptoms continued to progress. She is a *FMR1* premutation carrier with 88 *FMR1* CGG repeats. Her son has intellectual disability but has not been tested for FXS. Her father had severe tremor and ataxia. Her sister has fragile X-associated premature ovarian insufficiency (FXPOI). She has a male cousin with FXTAS and three female cousins who are also premutation carriers. Based on her MRI as well as ataxia and tremor, she met criteria for definite FXTAS.

### Case 18

An 80 year-old man presented with a three-year history of worsening falls. He had developed shuffling gait, a soft voice, was choking on food, and had a masked facial expression. He denied tremor. He had been diagnosed by a prior neurologist with Parkinson disease and started on 3.5 tablets of 25–100 mg carbidopa/levodopa. By report, his symptoms did not improve. On examination at the age of 80, his MMSE was 28/30. He had a positive glabellar reflex and negative applause sign. He had impaired vertical gaze, impaired vertical optokinetic nystagmus, and slowed saccades. He had symmetrically increased tone in both upper extremities and bradykinesia in all four extremities. He had left sided shoulder elevation with a mild rightward head turn. He had mild rest tremor in the left hand and mild kinetic tremor when drawing spirals. There was no evidence of dysmetria. He had a positive pull test, was unable to perform tandem gait, and took multiple steps to turn. His steps were slow and short. He was unable to have a MRI, but brain CT showed ventriculomegaly. He was started on 5 mg twice daily of memantine given complaints of poor memory, however this was subsequently discontinued as it proved ineffective. His falls initially decreased in frequency after physical therapy. Within 8 months of presentation, he was unable to walk on his own, even with a walker. He had a retrial of carbidopa/levodopa 25–100 mg three pills daily. His bradykinesia improved mildly, but he began experiencing delusions and hallucinations. The psychosis improved and entacapone 200 mg three times daily was added. The patients' family felt the entacapone helped the speech problems, but not the other motor features. He was a *FMR1* premutation carrier with 68 *FMR1* CGG repeats. His grandson had FXS. He met criteria for probable FXTAS given his tremor and ataxia, but lack of a MRI.

## Discussion

These cases have some similarities and differences to previously reported FXTAS phenotypes. The majority of the patients have a mixed movement disorder, with signs of tremor, ataxia, and parkinsonism. In addition, neuropathic findings and neuropsychiatric issues are common, with many having cognitive deficits on presentation. Our cases were found primarily in families with known fragile X-associated disorders. All of these features have been previously described.

Case 12 describes a woman with a strong family history of *FMR1* mutation associated diseases. Her course began with gait difficulty and evolved to include eye movement abnormalities consistent with progressive supranuclear palsy (PSP). In Case 18, the patient's history of falls at symptom onset, absence of response to carbidopa/levodopa, and upgaze palsy is most consistent diagnostically with PSP. This is a neurodegenerative movement disorder characterized by early falls, supranuclear ophthalmoplegia (particularly of vertical eye movements), parkinsonism, and later cognitive decline. In addition, several of the other cases in Table 1 had eye findings often seen in PSP, including decreased optokinetic nystagmus, especially in the vertical direction, slowed vertical saccades, and the presence of square wave jerks (Litvan et al., 1996). Case 2 demonstrated lack of vertical optokinetic nystagmus. Cases 13 and 17 demonstrated saccades which were hypometric and slow, respectively. A PSP phenotype has not been reported in FXTAS in the past. Schrag et al. (2006) estimated the prevalence of PSP in the general population at about 6 per 100,000. The prevalence of FXTAS has been reported at 1/4000 in men over 55 (Hall and Jacquemont, 2010). Given the rarity of these two disorders, it may be more than coincidence that five individuals in our series had a PSP-like phenotype.

It is unclear in our patients whether the PSP-like phenotype is a variation of FXTAS, whether this represents the presence of PSP in *FMR1* premutation carriers, or whether these patients have two neurodegenerative disorders. Most likely, the pathways involved with the classic eye findings and falls in PSP located in the brainstem are also involved in FXTAS, resulting in similar phenotypic presentations. However, previous case series have shown that *FMR1* premutation carriers may have dual pathology on autopsy, specifically in cases of FXTAS and Alzheimer disease, and suggest that they may be synergistic in creating a worse neurological phenotype (Tassone et al., 2012a). Autopsy on our cases will help to clarify this issue. The typical neuropathology associated with FXTAS is enlarged, inclusion-bearing astrocytes in the cerebral white matter and intra-nuclear inclusions in both the brain and spinal cord (Greco et al., 2006). This differs from the neuropathological findings seen in PSP, in particular neurofibrillary tangles and/or neutrophil threads in the striatum, substantia nigra, occulomotor complex, peri-aqueductal gray, superior colliculi, basis pontis, dentate nucleus, and prefrontal cortex (Litvan, 2005). Other abnormal and non-specific eye movement abnormalities were common in our cases. These are not thought to be part of FXTAS, but this topic needs to be studied further to determine if these are unique features of FXTAS that could aid in clinical diagnosis.

Cases 7 and 9 illustrate a more rapid progression of neurological signs commonly seen in FXTAS due to other spinal issues. The quicker deterioration might be due to a cumulative effect of a spinal cord problems and long tract dysfunction related to the premutation, which lead to a double hit of pathology in the crossing cerebellar fibers in the middle cerebellar peduncle (from the underlying FXTAS) and the afferent spinocerebellar fibers that travel up the spinal cord. In both cases, rapid diagnosis and aggressive rehabilitation were sufficient to improve the outcome of the acute worsening of the gait disorder in these patients. Clinicians should be alerted to monitor abrupt changes in the gait symptoms in FXTAS patients as this may be improved back to baseline if an underlying cause is found. It is also important to note that, like our patient, many FXTAS patients benefit from physical therapy, and thus it should be included as an integral part of their treatment.

Interestingly, the FXTAS Rating Scale scores did not appear to coincide with the number of CGG repeats or the certainty of diagnosis. Patients with “definite” FXTAS had FXTAS Rating Scale scores as low as 30 and as high as 75, with a range of CGG repeat between 74 and 124. Patients whose diagnosis was “probable” FXTAS had FXTAS Rating Scale Scores between 17 and 81, with repeat sizes that ranged from 55 to 150. “Possible” FXTAS patients had rating scales that ranged from 7 to 41, with CGG repeat sizes ranging from 85 to 120. The discrepancy between FXTAS rating scale and certainty of diagnosis may be explained by the fact that the diagnostic criteria of FXTAS require radiological signs on MRI for a definite FXTAS diagnosis and some of our patients were unable to have MRI imaging. This would have lowered the certainty of diagnosis in those cases.

A final observation seen is that the women who meet criteria for FXTAS had milder movement disorders than the men. Several published case series have suggested that the phenotype may be milder in women due to X-inactivation and the presence of a normal *FMR1* allele. With the exception of one female case in Table 1, this was also the case in our group of patients.

## Concluding remarks

This case series illustrates some new observations in patients with FXTAS. It also confirms the heterogeneity of the FXTAS phenotype which has previously been described, even in cases when the diagnosis is considered “definite.” The phenotype may include a PSP-like presentation. Rapid progression of the gait ataxia may be secondary to another spinal cord issue and should be investigated. In addition, the FXTAS Rating Scale score may not be informative regarding diagnosis. Additional studies in FXTAS cohorts, including autopsy, are needed to confirm these findings. The clinical heterogeneity of this disorder has implications for clinical trials in FXTAS, making outcomes based on clinical phenotypes alone less practical.

### Conflict of interest statement

The authors declare that the research was conducted in the absence of any commercial or financial relationships that could be construed as a potential conflict of interest.

## References

[B1] Adams J. S. Adams P. E. Nguyen D. Brunberg J. A. Tassone F. Zhang W.. (2007). Volumetric brain changes in females with fragile X-associated tremor/ataxia syndrome (FXTAS). Neurology 69, 851–859. 10.1212/01.wnl.0000269781.10417.7b 17724287

[B2] Apartis E. Blancher A. Wassilios G. M. Guyant-Marechal L. Maltete D. De Brouker T.. (2012). FXTAS: new insights and the need for revised diagnostic criteria. Neurology 79, 1898–1907. 10.1212/WNL.0b013e318271f7ff 23077007

[B3] Berry-Kravis E. Abrams L. Coffey S. M. Hall D. A. Greco C. Gane L. W.. (2007a). Fragile X-associated tremor/ataxia syndrome: clinical features, genetics and testing guidelines. Mov. Disord. 22, 2018–2030. 10.1002/mds.21493 17618523

[B4] Berry-Kravis E. Goetz C. G. Leehey M. A. Hagerman R. J. Zhang L. Li L.. (2007b). Neuropathic features in fragile X premutation carriers. Am. J. Med. Genet. 143A, 19–26. 10.1002/ajmg.a.31559 17152065

[B5] Bourgeois J. A. Farzin F. Brunberg J. A. Tassone F. Hagerman P. Zhang L.. (2006). Dementia with mood symptoms in a fragile X premutation carrier with the fragile X-associated tremor/ataxia syndrome: clinical intervention with donepezil and venlaflaxine. J. Neuropsychiatry Clin. Neurosci. 18, 171–177. 10.1176/appi.neuropsych.18.2.171 16720793

[B6] Coffey S. M. Cook K. Tartaglia N. Tassone F. Nguyen D. Pan R.. (2008). Expanded clinical phenotype of women with the *FMR1* premutation. Am. J. Med. Genet. 146A, 1009–1016. 10.1002/ajmg.a.32060 18348275 PMC2888464

[B7] Folstein M. F. Folstein S. E. McHugh P. R. (1975). “Mini-mental state”: a practice method for grading the cognitive state of patients for the clinician. J. Psychiatr. Res. 12, 189–198. 10.1016/0022-3956(75)90026-6 1202204

[B8] Greco C. M. Berman R. F. Martin R. M. Tassone F. Schwartz P. H. Chang A.. (2006). Neuropathology of fragile X-associated tremor/ataxia syndrome (FXTAS). Brain 129, 243–255. 10.1093/brain/awh683 16332642

[B9] Grigsby J. Brega A. G. Leehey M. A. Goodrich G. K. Jacquemont S. Loesch D. Z.. (2007). Impairment of executive cognitive functioning in males with fragile X-associated tremor/ataxia syndrome. Mov. Disord. 15, 645–650. 10.1002/mds.21359 17266074

[B10] Hall D. Jacquemont S. (2010). The Epidemiology of FXTAS. The Fragile X-associated Tremor/Ataxia Syndrome (FXTAS). New York, NY: Springer, 17–30

[B11] Jacquemont S. Hagerman R. J. Leehey M. A. Grigsby J. Zhang L. Brunberg J.. (2003). Fragile X premutation tremor/ataxia syndrome: molecular, clinical, and neuroimaging correlates. Am. J. Hum. Genet. 72, 869–878. 10.1086/374321 12638084 PMC1180350

[B12] Jacquemont S. Hagerman R. J. Leehey M. Hall D. A. Levine R. A. Brunberg J. A.. (2004). Penetrance of the fragile X-associated tremor/ataxia syndrome in a premutation carrier population. JAMA 291, 460–469. 10.1001/jama.291.4.460 14747503

[B13] Leehey M. (2009). Fragile X-associated tremor/ataxia syndrome: clinical phenotype, diagnosis and treatment. J. Investig. Med. 57, 830–836. 10.231/JIM.0b013e3181af59c4 19574929 PMC2787702

[B14] Leehey M. A. Berry-Kravis E. Goetz C. G. Zhang L. Hall D. A. Li L.. (2008). FMR1 CGG repeat length predicts motor dysfunction in premutation carriers. Neurology 70(16 Pt 2), 1397–1402. 10.1212/01.wnl.0000281692.98200.f5 18057320 PMC2685188

[B15] Leehey M. A. Legg W. Tassone F. Hagerman R. J. (2011). Fibromyalgia in *fragile X mental retardation 1* gene premutation carriers. Rheumatology (Oxford). 50, 2233–2236. 10.1093/rheumatology/ker273 21926154 PMC3222847

[B16] Litvan I (2005). Atypical Parkinsonian Disorders. Totowa, NJ: Humana. 10.1385/159259834X

[B17] Litvan I. Agid Y. Caine D. Campbell G. Dubois B. Duvosin R. C.. (1996). Clinical research criteria for the diagnosis of progressive supranuclear palsy (Steele-Richardson-Olszewski syndrome): report of the NINDS-SPSP international workshop. Neurology 47, 1–9 10.1212/WNL.47.1.1 8710059

[B18] Louis E. Moskowitz C. Friez M. Amaya M. Vonsattel J. P. G. (2006). Parkinsonism, dysautonomia, and intranuclear inclusions in a fragile X carrier: a clinical-pathological study. Mov. Disord. 21, 420–425. 10.1002/mds.20753 16250026

[B19] Schrag A. Selai C. Quinn N. Lees A. Litvan I. Lang A.. (2006). Measuring quality of life in PSP. Neurology 67, 39–44. 10.1212/01.wnl.0000223826.84080.97 16832075

[B20] Seltzer M. M. Baker M. W. Hong J. Maenner M. Greenberg J. Mandel D. (2012). Prevalence of CGG expansions of the *FMR1* gene in a US population-based sample. Am. J. Med. Genet. 159B, 589–597. 10.1002/ajmg.b.32065 22619118 PMC3391968

[B21] Tassone F. Greco C. M. Hunsaker M. R. Seritan A. L. Berman R. F. Gane L. W.. (2012a). Neuropathological, clinical and molecular pathology in female fragile X premutation carriers with and without FXTAS. Genes Brain Behav. 11, 577–585. 10.1111/j.1601-183X.2012.00779.x 22463693 PMC3965773

[B22] Tassone F. Iong K. P. Tong T. H. Lo J. Gane L. W. Berry-Kravis E.. (2012b). *FMR1* CGG allele size and prevalence ascertained through newborn screening in the United States. Genome Med. 4, 100. 10.1186/gm401 23259642 PMC4064316

